# Electrochemical Corrosion Behavior and Mechanism of (Al_x_)Mo_0.8_NbTi_y_W_0.2_Zr Refractory High-Entropy Alloys with Different Al Contents in NaCl Solution

**DOI:** 10.3390/ma19214535

**Published:** 2026-10-28

**Authors:** Bojun Zhao, Hongping Zhao, Guoqing Chen, Fan Yang, Yue Guo

**Affiliations:** 1 School of Mechan-Electronic Engineering, Suzhou Polytechnic University, Suzhou 215104, China; zbjjob@163.com (B.Z.); 92419@jssvc.edu.cn (Y.G.); 2 School of New Energy Engineering and Automotive, Huzhou Vocational and Technical College, Huzhou 313000, China; yangfan8008@163.com; 3 School of Materials Science and Engineering, Dalian University of Technology, Dalian 116085, China

**Keywords:** refractory high-entropy alloy, Al alloying, electrochemical corrosion, passivation, local corrosion

## Abstract

Refractory high-entropy alloys (RHEAs) are promising candidates for next-generation high-temperature structural applications because of their excellent high-temperature mechanical properties and environmental stability. However, in marine power systems and high-salinity environments, chloride-induced localized corrosion remains a key factor limiting their application. Although Al alloying is effective for weight reduction and microstructural regulation, its effects on the passivation behavior and corrosion mechanism of RHEAs remain unclear. In this work, the electrochemical corrosion behavior of (Al_x_)Mo_0.8_NbTi_y_W_0.2_Zr RHEAs with different Al contents in 3.5 wt.% NaCl solution was investigated, together with the corresponding post-corrosion microstructural evolution. All alloys exhibited clear passivation during potentiodynamic polarization, and the combined electrochemical results and thermodynamic considerations suggest that Nb-containing oxides may contribute to passivation in this alloy system. As the Al content increased, the passivation potential changed only slightly, whereas the passive-film breakdown-related potential decreased markedly, indicating reduced passive film stability. Microstructural analysis showed that Al-for-Ti substitution was associated with the formation of Al-Zr-rich compounds and an increase in their measured area fraction. The preferential dissolution of the Al-Zr-rich compounds during anodic polarization indicates their greater local corrosion susceptibility under the applied test conditions, while their spatial distribution suggests a contribution from microstructural electrochemical heterogeneity. Thus, the corrosion behavior of this alloy system in chloride-containing environments may reflect the combined effects of passivation, potentially involving Nb-containing oxides, and local electrochemical heterogeneity associated with the coupled increase in Al and decrease in Ti. Since Al and Ti contents are coupled (x + y = 2), the observed microstructural evolution and corrosion degradation cannot be attributed solely to Al, but rather to the synergistic effect of Al increment and Ti decrement.

## 1. Introduction

In complex service environments such as marine propulsion systems, offshore platforms, and aero-engines, critical hot-end components are required not only to withstand prolonged exposure to high temperatures, high stresses, and cyclic loading, but also to resist corrosion–oxidation synergistic damage induced by salt spray, seawater deposits, and chloride-containing media [1,2]. With the continuing demand for higher thrust-to-weight ratios, higher service temperatures, and longer lifetimes in advanced power equipment, the development of novel structural materials combining high-temperature load-bearing capability, microstructural stability, and corrosion resistance has become a key issue in the field of advanced engineering materials [3,4].

Refractory high-entropy alloys (RHEAs), which are composed of multiple high-melting-point elements, generally exhibit excellent high-temperature strength, high thermal stability, and considerable flexibility in compositional design, and are therefore regarded as important candidates for next-generation high-temperature structural materials [4,5,6,7,8,9,10,11]. Unlike conventional single-principal-element alloys, high-entropy alloys develop complex and tunable phase structures through multi-principal-element interactions, and their properties are jointly influenced by the high-entropy effect, lattice distortion, sluggish diffusion, and cocktail effect [5,6,7,12,13,14,15]. In addition to mechanical and thermal stability, these features also strongly affect surface passivation and corrosion response [16,17,18]. Previous studies have shown that some high-entropy alloys containing Cr, Ni, Mo, Al, and Ti can form relatively stable passive films in corrosive media and exhibit promising corrosion resistance in acidic and chloride-containing environments [13,16,17,19,20,21].

However, compared with the extensive literature on mechanical properties, the corrosion behavior of RHEAs remains far less systematically understood [13,16,17,21]. This is largely because such alloys usually possess complex multiphase microstructures, pronounced elemental partitioning, and strong local electrochemical heterogeneity. Their corrosion behavior depends not only on the overall composition, but also on phase constitution, elemental segregation, interphase characteristics, and the composition of the surface film [16,17,21,22]. In chloride-containing environments, the formation, breakdown, and repair of passive films are in constant competition. On the one hand, oxide films formed by high-valence metallic elements can enhance passivation; on the other hand, potential differences between constituent phases and their tendency toward selective dissolution can trigger galvanic corrosion and local film rupture, thereby promoting pitting or interphase-boundary-assisted localized corrosion [23,24]. Therefore, the corrosion resistance of multiprincipal, multiphase RHEAs is essentially governed by the competition between surface passivation capability and microstructural electrochemical heterogeneity.

As a typical lightweight alloying element, Al is widely used in high-temperature alloys and high-entropy alloys for reducing density and improving oxidation resistance [25,26,27,28]. In RHEAs, the addition of Al not only significantly lowers density, but also influences mechanical properties and high-temperature oxidation behavior by altering crystal structure and phase constitution, for example through promoting the formation of BCC/B2-related phases or intermetallic compounds [25,28]. From the corrosion perspective, however, the role of Al is distinctly dual in nature. A moderate amount of Al may improve surface film composition and enhance local passivation, whereas Al-induced second-phase precipitation, phase separation, and compositional fluctuations may increase electrochemical heterogeneity, intensify microgalvanic corrosion, and reduce overall corrosion resistance in chloride-containing media [29]. Thus, the effect of Al alloying on corrosion resistance is not unidirectional, but highly dependent on phase evolution and the electrochemical response of individual phases.

At present, research on Al-containing RHEAs has mainly focused on density reduction, phase regulation, high-temperature mechanical properties, and oxidation resistance, whereas their corrosion behavior in simulated seawater environments has received much less attention [8,9,10,29,30]. Although the corrosion behavior of several Al-containing high-entropy and refractory alloy systems has been investigated, the corrosion response of Mo-Nb-Ti-W-Zr-based refractory alloys under coupled Al-for-Ti substitution remains insufficiently understood. In particular, the relationships among the coupled increase in Al and decrease in Ti, the formation and distribution of Al-Zr-rich compounds, and localized corrosion in chloride-containing solutions have not been systematically clarified. In addition, the distribution of post-polarization corrosion damage among the constituent phases and its propagation into the subsurface remain unclear.

Therefore, in this work, (Al_x_)Mo_0.8_NbTi_y_W_0.2_Zr RHEAs with x + y = 2 were investigated in 3.5 wt.% NaCl solution using electrochemical measurements, same-area quasi-in-situ SEM observations, and FIB cross-sectional characterization. The relationships among the coupled Al-for-Ti substitution, phase evolution, electrochemical response, and spatial distribution of corrosion damage were examined. It should be noted that phase-resolved corrosion-potential differences and the chemical composition of the passive film were not directly measured in this study. Accordingly, the corresponding mechanisms are discussed as interpretations consistent with, but not uniquely demonstrated by, the available experimental evidence.

## 2. Materials and Methods

### 2.1. Alloy Preparation

In this study, Mo, Nb, W, Ti, and Zr were selected as the principal refractory elements, and Al was introduced as a lightweight alloying element to prepare (Al_x_)Mo_0.8_NbTi_y_W_0.2_Zr alloys with different Al contents, where x + y = 2. The raw materials were commercially available high-purity micrometer-sized metal powders (Aladdin Biochemical Technology Co., Ltd., Shanghai, China). Except for Zr powder with a purity of 99.5%, all other powders had a purity of 99.9%, and the average particle size of each powder was no larger than 45 μm.

Before ball milling, the elemental powders were weighed according to the designed compositions using an electronic balance (MH-05) with a precision of 0.0001 g, and the weighing error was controlled within ±0.0005 g. The mixed powders were then loaded into a stainless-steel vacuum milling jar, followed by at least three vacuuming and backfilling cycles with high-purity Ar in order to minimize the residual air content inside the jar. Mechanical alloying was carried out using a planetary high-energy ball mill (QM-3SP4, Nanjing University Instrument Co., Ltd., Nanjing, China). Tungsten carbide balls were used as the milling media, with a ball-to-powder weight ratio of 10:1 and a rotational speed of 400 r/min. The milling process was conducted in an intermittent mode, i.e., 60 min of milling followed by a 15 min pause, and the total milling time was 42 h.

**Figure 1:**
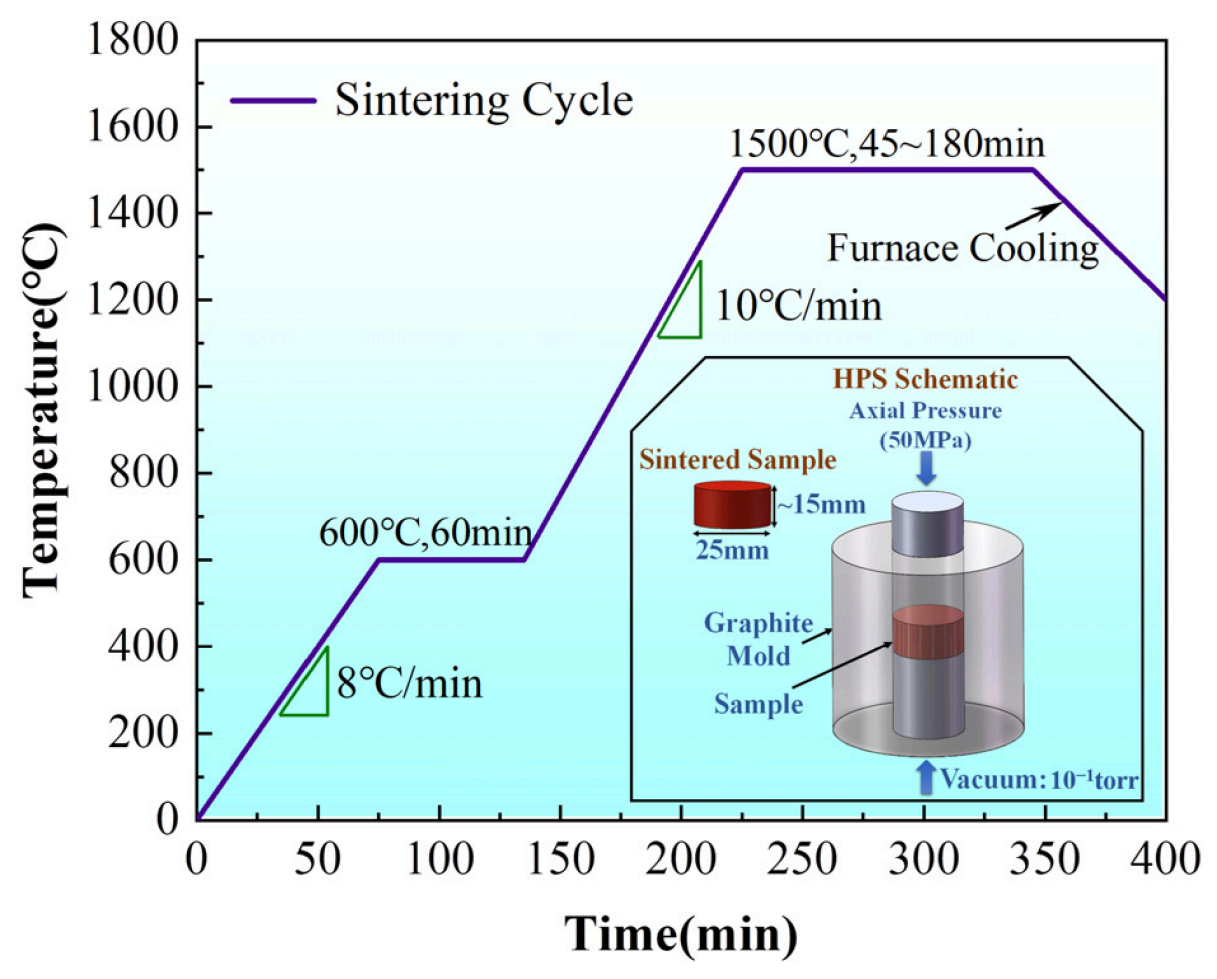
The profile of sintering process for powders and the schematic diagram of sintering mold structure.

The ball-milled alloy powders were consolidated into bulk samples by vacuum hot-press sintering using a ZRY-55 hot-press furnace (Jinzhou Boda High Temperature Material Equipment Manufacturing Co., Ltd., Jinzhou, China). A schematic of the hot-press sintering route and die structure is shown in Figure 1. The powders were loaded into a cylindrical graphite die with an inner diameter of 25 mm. Graphite paper coated with sprayed nano-BN was placed at the powder/die interfaces to reduce reactions between the powders and the graphite die. Sintering was performed under a relative vacuum of approximately 10^−1^ torr. The samples were first heated from room temperature to 600 °C at a rate of 8 °C/min and held at this temperature for 60 min. They were subsequently heated to 1500 °C at a rate of 10 °C/min. At 1500 °C, the Al-0, Al-0.2, Al-0.5, and Al-1 alloys were held for 180, 150, 120, and 45 min, respectively, after which the samples were furnace-cooled to room temperature. The holding time was composition-dependent, whereas the sintering temperature and applied axial pressure were kept constant at 1500 °C and 50 MPa, respectively, for all compositions. The different holding times were selected to account for the composition-dependent consolidation behavior of the refractory alloy powders. For the low-Al alloys, longer high-temperature holding was used to provide sufficient time for diffusion-assisted particle bonding, pore elimination, and chemical homogenization. With increasing Al content, pressure-assisted consolidation and diffusion were considered more favorable, motivating the use of a shorter holding period. In addition, prolonged exposure of the Al-rich alloys at 1500 °C under vacuum was avoided to reduce the possible loss of Al and excessive microstructural coarsening. Therefore, the holding time was progressively reduced with increasing Al content. Axial pressure was gradually applied during the initial heating stage, increased to 50 MPa during the holding stage at 600 °C, and then maintained throughout the subsequent heating and high-temperature holding processes.

The bulk densities of the hot-pressed alloys were determined using the Archimedes method. At least 3 measurements were performed for each composition, and the results are reported as the mean ± standard deviation. The bulk densities of the Al-0, Al-0.2, Al-0.5, and Al-1 alloys were 7.99 ± 0.05, 7.51 ± 0.08, 7.45 ± 0.04, and 7.39 ± 0.02 g·cm^−3^, respectively.

### 2.2. Phase and Microstructure Characterization

The phase constitution of the sintered alloys was analyzed by X-ray diffraction (XRD, EMPYREAN, PANalytical, Almelo, The Netherlands). Cu Kα radiation (λ = 1.5406 Å) was used, with an operating voltage of 45 kV and a current of 45 mA. The scanning range was 20–100°, and the scanning rate was 4°/min. The diffraction data were analyzed using Jade 6.0 software together with the PDF2-2004 database for phase identification.

The microstructures of the alloys were characterized using a field-emission scanning electron microscope (FE-SEM, SUPER55-32760, Carl Zeiss, Berlin, Germany). Secondary electron (SE) and backscattered electron (BSE) images were collected to examine the microstructural features, phase distribution, and surface morphology before and after corrosion. Prior to observation, the specimens for microstructural characterization were ground successively using SiC papers, mechanically polished to a mirror finish, ultrasonically cleaned, and thoroughly dried.

The local chemical compositions of the constituent phases were measured by field-emission electron probe microanalysis (EPMA, JXA-8530F PLUS, JEOL, Tokyo, Japan). Different phases were identified in BSE mode, followed by point analysis to determine their compositions. The EPMA operating voltage was 5–15 kV, and quantitative analysis was performed using the ZAF correction method. Compositional analysis was conducted using five wavelength-dispersive spectrometers (WDS) equipped with different diffraction crystals (e.g., LiF, PET, TAP) to simultaneously detect the characteristic X-rays of Al, Mo, Nb, Ti, W, Zr, and O elements. Under an operating voltage of 15 kV, the electron beam penetrates approximately 1–3 μm into the material; at 5 kV, the analysis depth is reduced to a few hundred nanometers. Given the relatively small scale of the second phases, a lower operating voltage was preferred to minimize the matrix excitation effect. Because the measured composition is an average over the interaction volume, the EPMA point analysis provides a near-surface composition that reflects the overall phase composition but may slightly deviate from the bulk if significant elemental micro-segregation exists within the interaction volume. Considering the powder metallurgy and vacuum hot-press sintering route used, a relatively uniform composition from the surface through to the bulk is expected for each individual phase, although interphase elemental partitioning is pronounced.

### 2.3. Electrochemical Testing and Corrosion Morphology Characterization

The corrosion behavior of the sintered (Al_x_)Mo_0.8_NbTi_y_W_0.2_Zr alloys in 3.5 wt.% NaCl solution was investigated electrochemically at room temperature. Plate-like specimens with dimensions of 15 mm × 15 mm × 2 mm were cut from the sintered bulk samples by electrical discharge wire cutting. Prior to testing, the specimen surfaces were ground sequentially using SiC papers up to 3000 grit, polished to a mirror finish with diamond paste, rinsed with deionized water, degreased in absolute ethanol, and dried.

Electrochemical measurements were performed using a CorrTest CS310 electrochemical workstation (Wuhan Corrtest Instruments Corp., Ltd., Wuhan, China) in a conventional three-electrode cell. The alloy specimen served as the working electrode, a saturated calomel electrode (SCE) as the reference electrode, and a platinum mesh as the counter electrode. During testing, the working electrode was mounted using a copper conductive clamp and positioned vertically on one side of the electrolyte cell, with an exposed area of 1 cm^2^. Unless otherwise stated, all electrode potentials reported in this work are referenced to the SCE. The corrosive medium was a 3.5 wt.% NaCl solution prepared from analytical-grade NaCl and deionized water with a resistivity higher than 18.2 MΩ·cm.

Before each test, the specimen was immersed in the solution and stabilized at its open-circuit potential (OCP) for 60 min to allow the electrode/solution interface to reach a relatively steady state. Electrochemical impedance spectroscopy (EIS) measurements were performed at OCP using a sinusoidal perturbation amplitude of 10 mV over the frequency range from 10^5^ to 10^−2^ Hz. The impedance data were fitted using the ZView 2 module in CS Studio 5 software. Potentiodynamic polarization tests were conducted at a scan rate of 1 mV/s. The potential scan was initiated at OCP −0.5 V (vs OCP) and terminated at 2.0 V (vs OCP); thus, the scan limits were defined relative to the stabilized OCP. The potential values recorded and reported from the polarization curves, including Ep and Ept, are expressed relative to the SCE.

In this work, the passivation potential, Ep, was operationally defined as the potential at which the anodic current density first became nearly independent of potential upon entering the passive region. The potential Ept was determined as the potential at which the anodic current density increased sharply again after the passive region. Because this sharp current increase was accompanied by localized dissolution and pitting-like surface damage, Ept is referred to here as the passive-film breakdown potential or breakdown-related transition potential, rather than being regarded as definitive evidence of a true transpassive transition. These parameters were used to comparatively evaluate the formation and stability of the passive films under the applied forward anodic scan. Since no cyclic polarization or reverse scan was performed, the present measurements do not provide direct evidence of repassivation behavior.

To further reveal the local damage features after electrochemical corrosion and the inward propagation behavior along the thickness direction, both surface and cross-sectional morphologies of the corroded specimens were characterized. The post-corrosion surface morphology was observed by SEM to analyze local dissolution and preferential corrosion of different microstructural phases. To obtain depth-resolved information from the corroded regions, focused ion beam (FIB) milling was employed to prepare cross sections using a Helios G4 UX system (Thermo Fisher, Waltham, MA, USA). During sample preparation, a Pt protective layer with dimensions of approximately 30 μm × 6 μm × 3 μm was first deposited on the region of interest, followed by sectioning and fine polishing under the protection layer so as to preserve the original structure of the matrix and corroded region as much as possible. The cross-sectional morphology was finally observed at a tilt angle of 52° to analyze pit depth, local void formation, and corrosion penetration into the matrix.

## 3. Results and Discussion

### 3.1. Evolution of Alloy Microstructure

**Figure 2:**
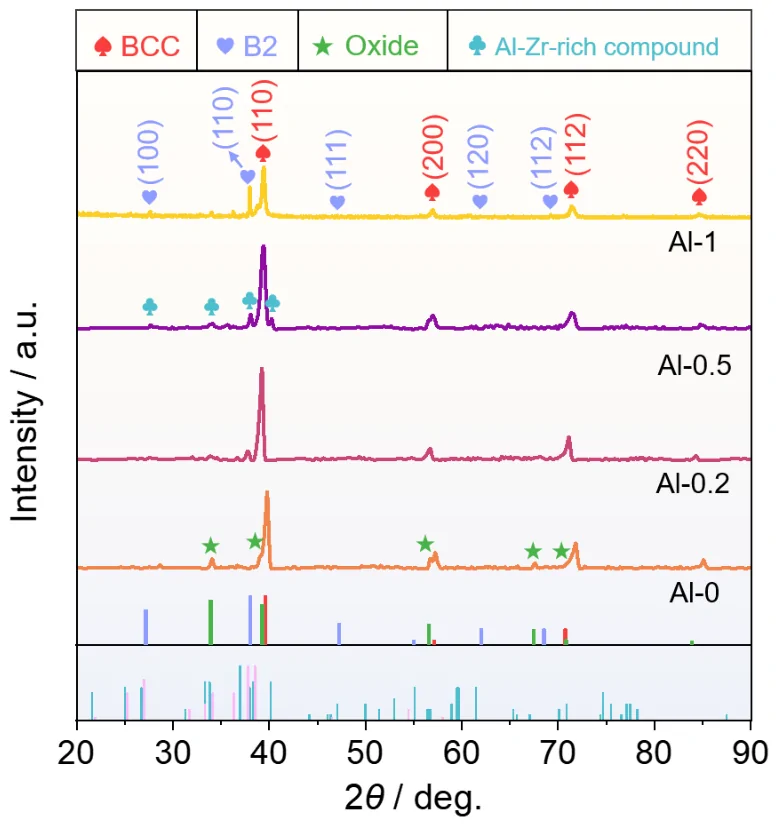
The XRD results and the PDF-card peaks of each phase of as-sintered alloys.

Figure 2 shows the XRD patterns of the sintered (Al_x_)Mo_0.8_NbTi_y_W_0.2_Zr alloys together with phase identification based on PDF reference cards. All alloys are dominated by diffraction peaks corresponding to the BCC phase, indicating that the BCC structure remains the matrix after sintering. In addition, several weak extra diffraction peaks are observed in each alloy, suggesting that minor secondary phases are formed besides the disordered BCC solid solution. For the Al-0 alloy, weak diffraction peaks corresponding to an FCC oxide phase are detected in addition to the BCC matrix. After the introduction of Al, new diffraction peaks appear in the Al-0.2 and Al-0.5 alloys, which can be assigned to Al-Zr intermetallic compounds with different atomic ratios, indicating that Al promotes the formation of Zr-rich compound phases during sintering. When the Al content is further increased to Al-1, distinct superlattice peaks characteristic of an ordered B2 phase emerge in addition to the Al-Zr intermetallic-related peaks, including (100)_B2_, (110)_B2_, and (112)_B2_. These results demonstrate that Al has a significant influence on phase constitution and ordering behavior in this alloy system. At low Al contents, the alloy remains mainly a disordered BCC solid solution accompanied by Al-Zr intermetallic precipitation, whereas with increasing Al content the ordering tendency of the BCC matrix becomes stronger and eventually leads to the formation of an identifiable B2 ordered structure in the Al-1 alloy.

**Figure 3:**
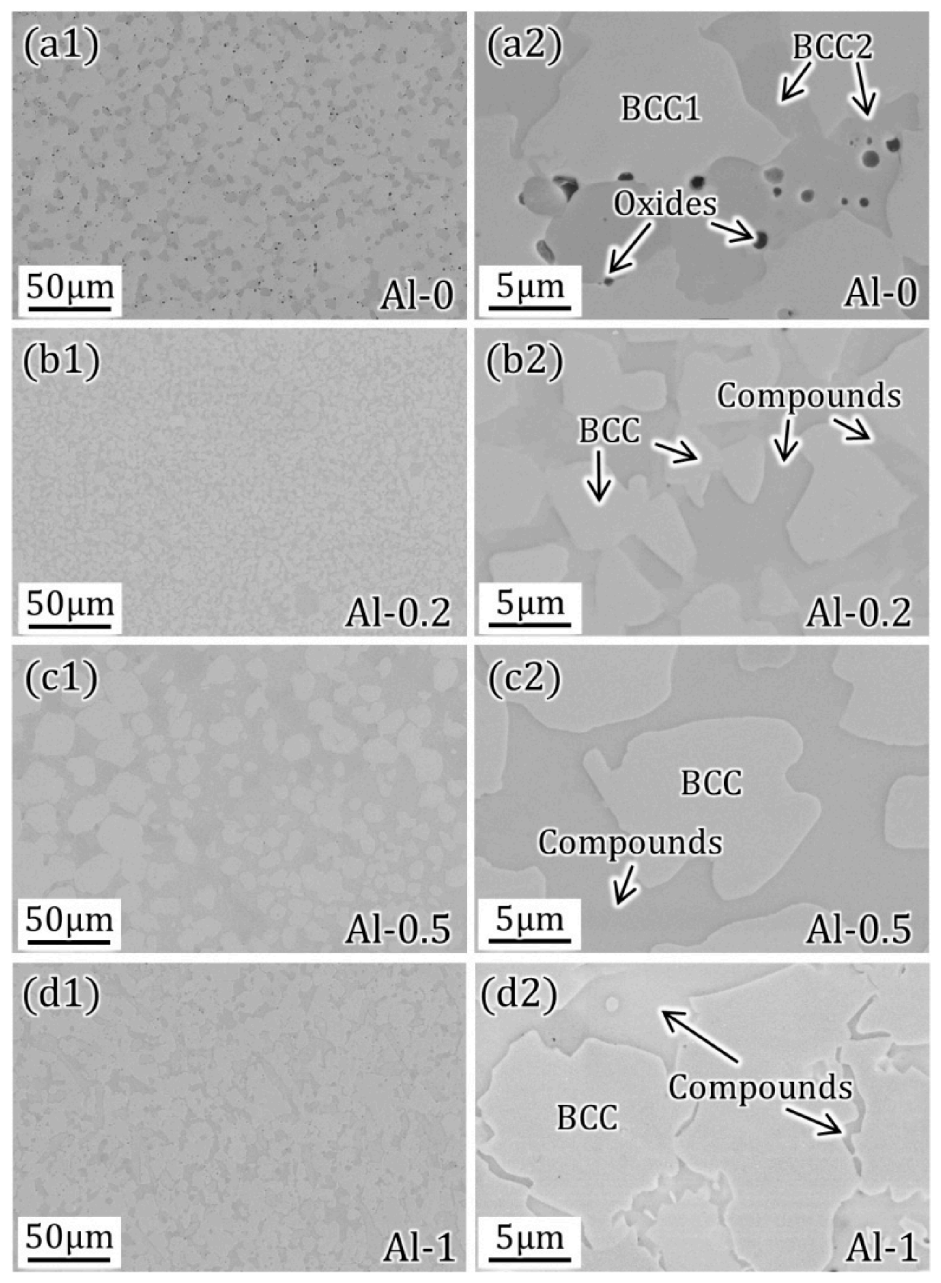
Microstructures of as-sintered (Al_x_)Mo_0.8_NbTiyW_0.2_Zr alloys: (**a1**–**d1**) are the low magnified BSE images while (**a2**–**d2**) are the high magnified BSE images.

Figure 3 shows the SEM microstructures of the sintered (Al_x_)Mo_0.8_NbTi_y_W_0.2_Zr alloys. All alloys exhibit a clearly multiphase microstructure, indicating that a single solid solution is not formed after sintering. Apart from the relatively obvious oxide phase present in the Al-0 alloy, the Al-containing alloys show similar general morphologies in BSE images, with the main differences lying in phase fraction and distribution. This suggests that the addition of Al does not significantly alter the overall morphological features of the microstructure, but directly affects phase constitution and elemental partitioning. In addition, no distinct morphological feature that can be unequivocally assigned to the B2 ordered phase is observed in the BSE image of the Al-1 alloy, implying that this phase may be too fine in scale or may not provide sufficient atomic-number contrast relative to the BCC matrix for direct discrimination under conventional BSE imaging.

The local chemical compositions of the constituent phases were determined by EPMA point analyses, and the results are summarized in Table 1. In contrast, the nominal bulk compositions of the investigated alloys, calculated from the initial powder stoichiometry and expressed in atomic percentages (at.%), are listed in Table 2. Direct bulk chemical analysis of the sintered alloys was not performed in this study. Therefore, Table 2 reports the nominal compositions based on the initial powder stoichiometry, whereas Table 1 reports local phase compositions measured by EPMA. The Al-0 alloy mainly consists of two BCC phases and one FCC oxide phase, in agreement with the XRD results. Starting from the Al-0.2 alloy, a small amount of O is detected in both the matrix and compound phases, but the O content is clearly higher in the compound phase, indicating that O preferentially enriches in the second-phase regions. In the Al-0.2 to Al-1 alloys, the dark-gray phases are all characterized by pronounced enrichment in Al and Zr and relatively low contents of the other elements, indicating that these phases are Al-Zr-based intermetallic compounds. The difference is that the corresponding phase in the Al-0.2 alloy also contains a certain amount of Ti, whereas two compositionally distinct Al-Zr-rich compound phases are present in the Al-1 alloy.

With increasing Al content, the formation mode of the second phase changes significantly. At low Al contents, the amount of Al available to combine with Zr is limited, and Ti also participates in compound formation, so that the second phase in the Al-0.2 alloy exhibits co-enrichment of Al, Zr, and Ti. With a further increase in Al accompanied by a decrease in Ti, the Ti content in the second phase gradually decreases, while Al-Zr enrichment becomes more pronounced, indicating that the compound phase evolves progressively toward more typical binary Al-Zr intermetallics. EPMA revealed Al- and Zr-enriched compound regions in the Al-0.5 and Al-1 alloys. Because the available data do not permit definitive identification of their exact stoichiometries, these regions are referred to here as Al-Zr-rich compounds. These results indicate that at high Al contents the alloy develops a complex multiphase microstructure consisting of BCC, B2, and multiple Al-Zr compounds, with more pronounced internal compositional fluctuations and a larger number of phase boundaries.

To quantitatively characterize the evolution of the Al-Zr-rich compound phases with composition, image analysis was performed using ImageJ 1.47 software based on the BSE micrographs. No Al-Zr-rich intermetallic compound was detected in the Al-0 alloy, whereas the corresponding area fraction was approximately 15.97% for Al-0.2, 22.83% for Al-0.5, and 27.86% for Al-1. These results demonstrate that the Al-for-Ti substitution progressively increases the amount of Al-Zr-rich compound phases. In addition to the increase in area fraction, the spatial distribution of these compounds changes from relatively discrete regions at low Al contents to more continuous and interconnected regions along matrix grain or interphase boundaries at higher Al contents. Because these Al-Zr-rich compounds are preferentially located at phase boundaries, their area fraction provides an indirect quantitative indication of the extent of second-phase enrichment associated with the grain/interphase-boundary regions.

The bulk densities of the Al-0, Al-0.2, Al-0.5, and Al-1 alloys were 7.99 ± 0.05, 7.51 ± 0.08, 7.45 ± 0.04, and 7.39 ± 0.02 g·cm^−3^, respectively. These values represent bulk densities rather than relative densities. Relative density and residual porosity were not quantified; therefore, differences in porosity and their possible contribution to localized corrosion cannot be excluded on the basis of these values alone. The observed increase in the amount and connectivity of the Al-Zr-rich compound phases nevertheless provides microstructural evidence relevant to the localized corrosion behavior. It should be noted that the holding time at 1500 °C decreased systematically with increasing Al-for-Ti substitution. This processing variable may have influenced diffusion, phase fraction, compound connectivity, grain structure, and chemical homogenization in addition to the effect of composition. Therefore, the observed increase in the amount and connectivity of the Al-Zr-rich compounds, as well as the associated corrosion evolution, should be interpreted as the combined result of composition and thermal-processing history. Because the holding time was not kept constant among the alloys, its independent contribution cannot be fully separated from that of the coupled Al increase and Ti decrease in the present study.

### 3.2. Electrochemical Corrosion Behavior

**Figure 4:**
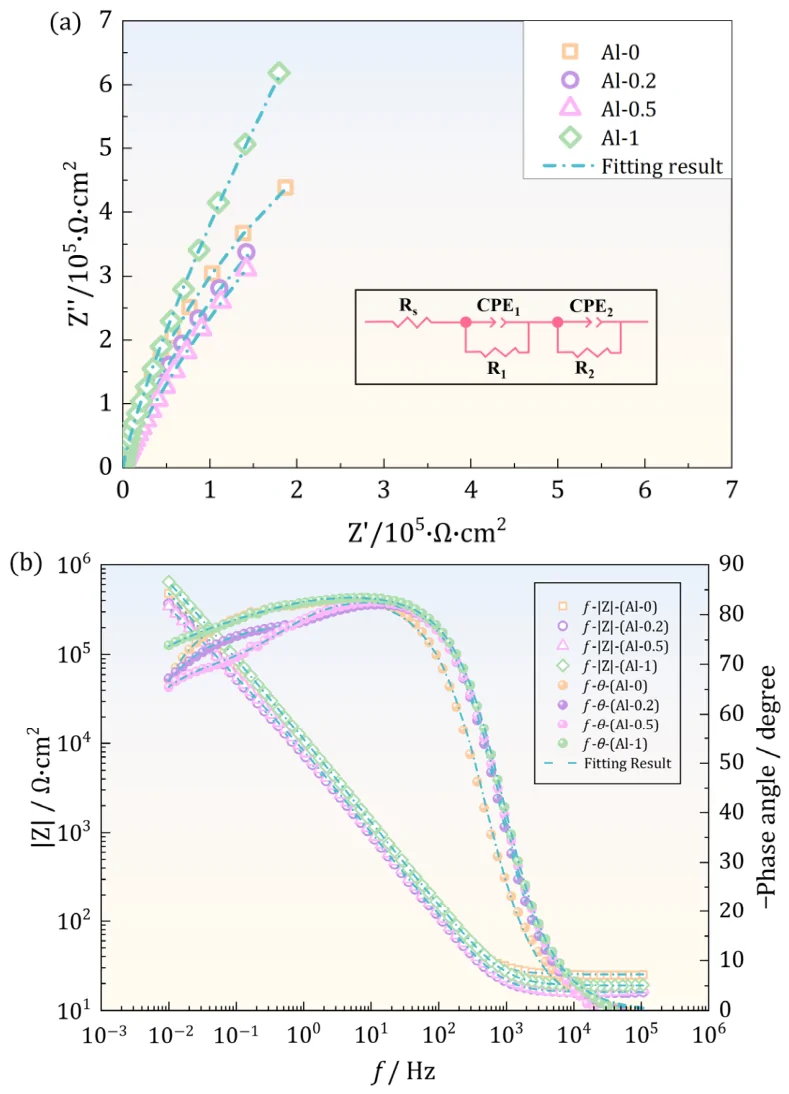

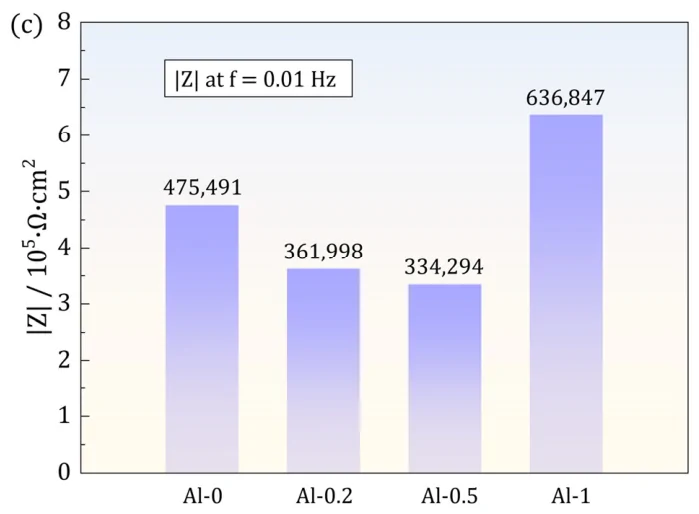
EIS results of (Al_x_)Mo_0.8_NbTi_y_W_0.2_Zr alloys in 3.5 wt.% NaCl: (**a**) Nyquist diagrams; (**b**) f-|z| and f-phase angle, respectively (polygons represent the experimental results and dotted lines represent the fitting results); (**c**) histogram of |z| at f = 0.01 Hz extracted from (**b**).

Based on the above microstructural analysis, EIS was further employed to evaluate the corrosion behavior of the (Al_x_)Mo_0.8_NbTi_y_W_0.2_Zr alloys in 3.5 wt.% NaCl solution. EIS can distinguish interfacial charge transfer, surface film response, and diffusion processes over a broad frequency range, and is therefore widely used to characterize the electrochemical behavior of material surfaces [31]. As shown in Figure 4a, the Nyquist plots of all samples display a predominantly capacitive response. The corresponding Bode plots exhibit a broad phase-angle response, which suggests the presence of two overlapping electrochemical relaxation processes [32]. However, this feature alone does not provide conclusive evidence for a physically duplex passive film or establish the existence of chemically distinct inner and outer layers.

To describe the measured impedance response, both a single-time-constant circuit, R_s_(CPER_ct_), and a two-time-constant circuit, R_s_(CPE_1_R_1_)(CPE_2_R_2_), were considered. The two-time-constant circuit was selected for parameterizing the impedance response and comparing the electrochemical behavior of the investigated alloys. Because a complete quantitative comparison of the residuals obtained from the two circuits is not available, the present study does not claim that the selected circuit is statistically superior to the single-time-constant circuit.

Accordingly, the circuit R_s_(CPE_1_R_1_)(CPE_2_R_2_) was used for comparative analysis. Here, R_s_ represents the solution resistance, whereas CPE_1_R_1_ and CPE_2_R_2_ represent two overlapping interfacial relaxation contributions. The higher-frequency response may be associated with relatively rapid processes involving the surface film/electrolyte interface, whereas the lower-frequency response may reflect slower barrier and charge-transfer processes involving the film/alloy interface and electrochemically heterogeneous regions. These assignments are tentative and are not intended to demonstrate the presence of two chemically distinct passive-film layers or a physically duplex film [33,34]. The fitted parameters are summarized in Table 3.

In general, the impedance modulus at low frequency, |Z|_f=0.01 Hz_, can be used as an indicator of the interfacial corrosion resistance [35]. The |Z| values at 0.01 Hz extracted from Figure 4b are summarized in Figure 4c. Combined with the fitted results, the addition of Al-for-Ti substitution significantly affects the impedance response of the alloy/electrolyte interface. Compared with the Al-0 alloy, the Al-0.2 alloy exhibits a lower low-frequency |Z| value, accompanied by decreases in both R_1_ and R_2_. These changes indicate a reduced impedance response and weakened interfacial barrier characteristics under the tested conditions. When the Al-for-Ti substitution increases to the Al-0.5 composition, the low-frequency impedance decreases further, suggesting a further reduction in the overall interfacial resistance response.

Under the tested open-circuit conditions, the Al-1 alloy exhibited a higher low-frequency impedance and larger fitted resistance values than the Al-0.2 and Al-0.5 alloys. This result indicates a stronger impedance response under these conditions, but does not by itself establish the chemical composition or long-term stability of the passive film. The two overlapping time constants may reflect contributions from the surface-film/electrolyte interface, the film/alloy interface, and the electrochemically heterogeneous multiphase microstructure. However, the present EIS data do not allow these contributions to be uniquely assigned to chemically distinct passive-film layers.

In combination with the above microstructural results, it can be seen that the effect of Al on corrosion behavior is not simply monotonic. At low Al contents, the increased microstructural heterogeneity and local electrochemical inhomogeneity at the second phase/matrix interfaces are detrimental to passive film formation and stability [36]; at higher Al contents, however, the beneficial effect of Al on surface film formation begins to emerge, thereby enhancing the barrier capability of the film under open-circuit conditions. Regarding the match between the proposed EIS model and the observed phase structures, the two overlapping time constants may reflect the surface-film/electrolyte interface, the film/alloy interface, and the underlying multiphase microstructure. However, their specific physical origins cannot be uniquely determined from the present EIS data. The high-frequency time constant (CPE1-R1) may be associated with relatively rapid interfacial processes, whereas the low-frequency time constant (CPE2-R2) may reflect slower barrier or charge-transfer processes involving the film/alloy interface and heterogeneous regions. These assignments remain tentative. For the Al-0 alloy, the presence of the BCC2 phase and oxides creates microstructural heterogeneity, leading to localized variations in film thickness and properties. With a further increase in Al accompanied by a decrease in Ti, the formation of Al-Zr intermetallic compounds at grain boundaries introduces strong electrochemical heterogeneity. These second-phase regions show preferential dissolution after polarization, indicating greater local susceptibility under the applied test conditions; their phase-specific corrosion potentials and passive-film thicknesses were not directly measured. Consequently, the decreased R_2_ values in the Al-0.2 and Al-0.5 alloys effectively capture the impaired barrier property at these localized heterogeneity sites, demonstrating a good agreement between the EIS response and the spatial distribution of the observed multiphase microstructure.

**Figure 5:**
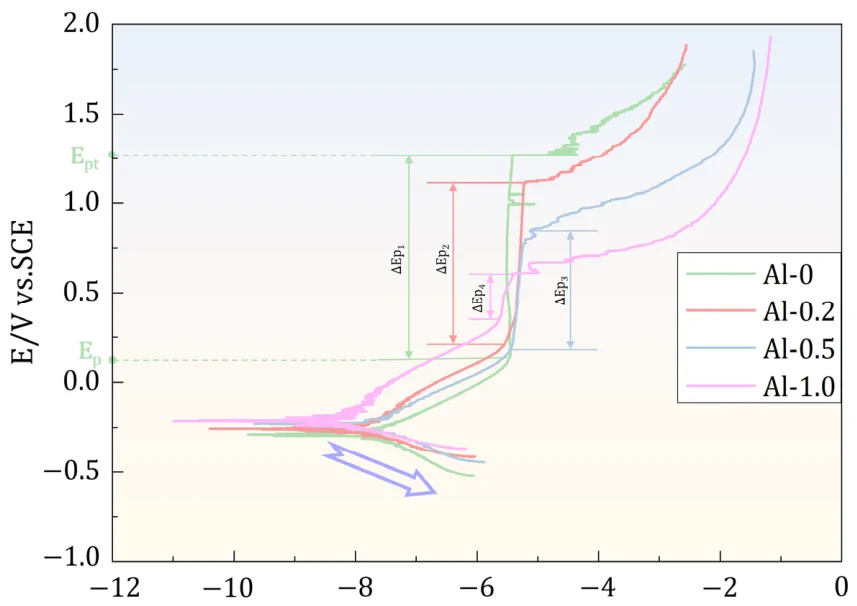
Potentiodynamic polarization curves of the Al-0, Al-0.2, Al-0.5, and Al-1 alloys in 3.5 wt.% NaCl solution. The scans were initiated at −0.5 V (vs OCP) and terminated at 2.0 V (vs OCP). All potentials are reported versus the saturated calomel electrode (SCE).

Compared with EIS, potentiodynamic polarization is more effective for evaluating the stability of the surface film and the susceptibility to localized corrosion under an externally applied anodic potential. Figure 5 presents the potentiodynamic polarization curves of the Al-0, Al-0.2, Al-0.5, and Al-1 alloys in 3.5 wt.% NaCl solution. The polarization scan was conducted from −0.5 V (vs OCP) to 2.0 V (vs OCP), whereas all potentials shown in the figure are reported relative to the SCE.

In the present study, Ep was identified as the potential at which the anodic current density first became approximately independent of potential upon entering the passive region. The parameter Ept was identified as the potential at which the anodic current density increased sharply again after the passive region. Although Ept is conventionally sometimes referred to as the transpassive potential, the sharp current increase observed in the present study was accompanied by localized dissolution and pitting-like surface damage. Therefore, Ept is interpreted here as a passive-film breakdown or localized-dissolution-related transition potential, rather than as definitive evidence of a true transpassive reaction. The passivation interval, ΔEp, was calculated as the difference between Ept and Ep:

Δ*Ep* = *Ept* − *Ep*

**Figure 6:**
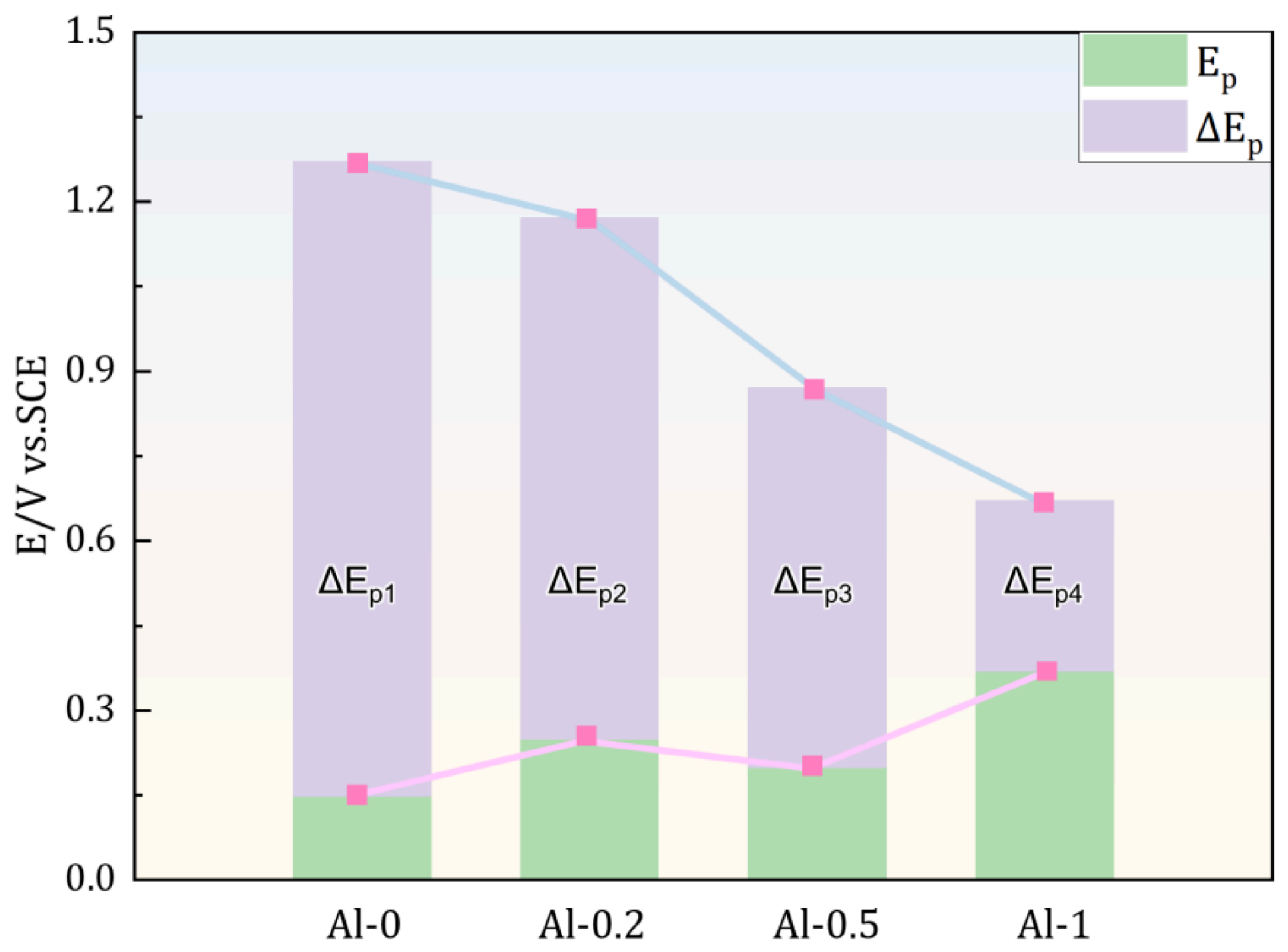
Values of the passivation potential (Ep) and passive-film breakdown-related potential (Ept), together with the corresponding passivation interval (ΔEp = Ept − Ep), for the Al-0, Al-0.2, Al-0.5, and Al-1 alloys derived from the potentiodynamic polarization curves. All potentials are reported versus the SCE.

Although ideal Tafel-linear regions were not clearly observed in the polarization curves, making accurate extrapolation of the corrosion rate difficult, the overall comparative trend was evident. With increasing Al-for-Ti substitution, the corrosion potential shifted in the negative direction and the anodic current density generally increased, indicating a higher corrosion tendency under the present testing condition. All alloys exhibited a passive-like region during the forward anodic scan. However, Ept decreased continuously with increasing Al content, while Ep also showed an overall decreasing tendency. Consequently, ΔEp decreased from approximately 1.1 V for the Al-0 alloy to 0.3 V for the Al-1 alloy (Figure 6). This result indicates that increasing Al-for-Ti substitution narrows the potential range over which the alloys maintain a relatively low anodic current density and promotes earlier passive-film breakdown or localized dissolution.

It should be emphasized that the forward potentiodynamic scans used in this study do not permit a direct evaluation of repassivation behavior. Such an evaluation would require cyclic polarization, reverse scanning, or potentiostatic measurements after localized breakdown. Therefore, the present results are limited to the comparative assessment of passive-film formation and breakdown under the applied forward anodic polarization.

Additionally, EIS can provide preliminary insights into distinguishing uniform dissolution from selective or localized corrosion. In a uniform dissolution process, the impedance response typically exhibits a single well-defined time constant with relatively high phase angles over a broad frequency range, reflecting a homogeneous charge-transfer process across the entire electrode surface. In contrast, localized or selective dissolution introduces localized breakdown of the passive film and the formation of active dissolution pits, which often manifests as an additional time constant in the low-frequency region or a phenomenon analogous to finite-length diffusion (e.g., inductive loops or Warburg impedance). In the present study, although the EIS results of all alloys display a single capacitive arc, the broad phase-angle peak (Figure 4b) suggests overlapping relaxation processes, but does not establish a duplex film structure. At low Al contents, the decrease in low-frequency impedance modulus primarily reflects a reduction in the overall barrier property of the passive film, tending toward uniform deterioration. However, this near-equilibrium EIS response is less sensitive to the early stages of selective dissolution at phase boundaries. Therefore, while EIS can qualitatively indicate the tendency toward localized breakdown via low-frequency impedance drops, the precise identification of selective phase dissolution in this alloy system relies more effectively on the combination of potentiodynamic polarization and quasi-in situ SEM/FIB cross-sectional observations.

**Figure 7:**
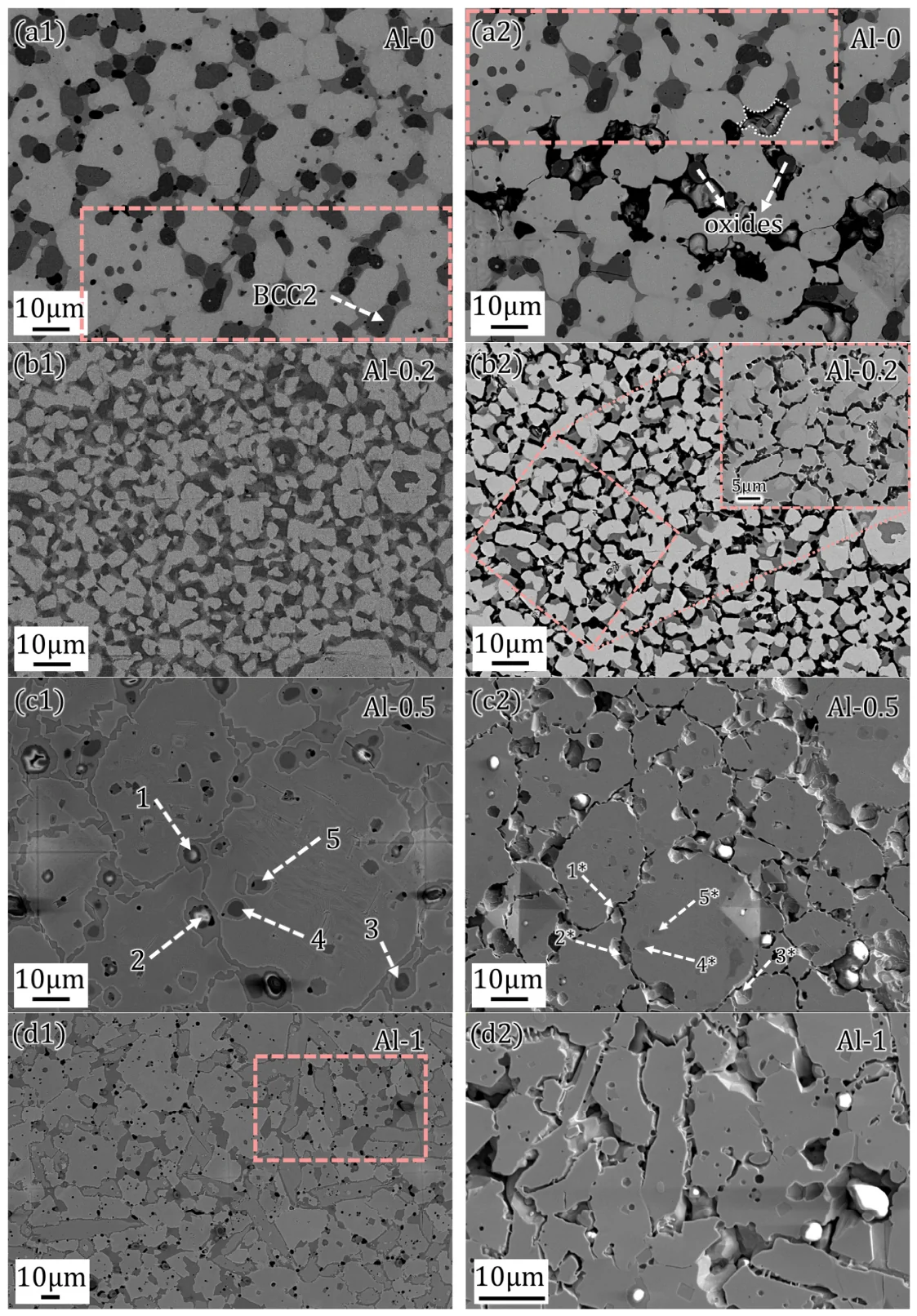
SEM morphology of Al-0, Al-0.2, Al-0.5 and Al-1 alloys before and after potential-dynamic testing: (**a1**,**a2**) Al-0; (**b1**,**b2**) Al-0.2; (**c1**,**c2**) Al-0.5 (1–5 and 1*–5* represent the in situ morphologies at the corresponding positions before and after the electrochemical test, respectively.); (**d1**,**d2**) Al-1. (The dotted-line boxes in (**a1**,**a2**) show the morphologies of the same region before and after the electrochemical test, respectively; (**d2**) shows the morphology of the dotted-line box in (**d1**) after the electrochemical test).

**Figure 8:**
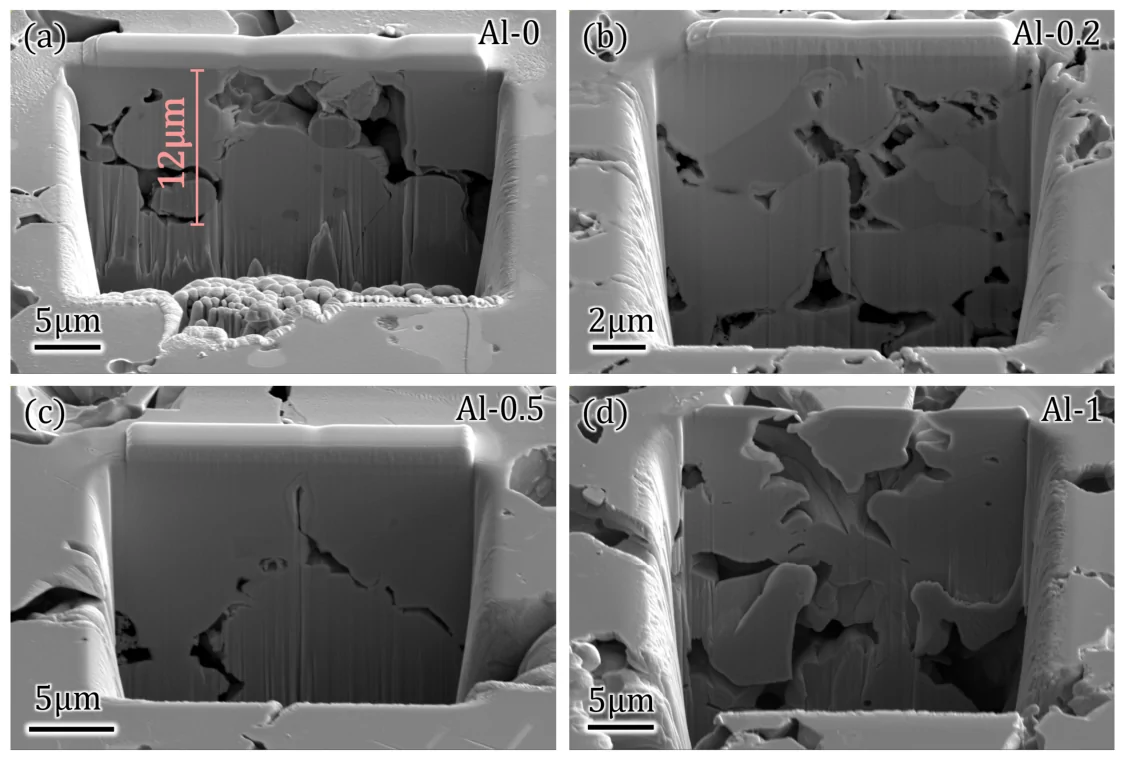
Cross-sectional morphology of Al-0, Al-0.2, Al-0.5 and Al-1 alloys after potentiodynamic testing obtained by FIB processing: (**a**) Al-0; (**b**) Al-0.2; (**c**) Al-0.5; (**d**) Al-1.

To further clarify the localized corrosion behavior and preferential dissolution sequence of different microstructural phases during the potentiodynamic scan, quasi-in situ SEM observations of the same region before and after corrosion were performed, together with FIB cross-sectional characterization of the corroded regions, as shown in Figure 7 and Figure 8, respectively. According to the comparison of the same area before and after corrosion in Figure 7(a1,a2), together with the phase compositions listed in Table 1, it can be concluded that in the Al-0 alloy the BCC2 phase is preferentially dissolved after the potentiodynamic scan, whereas the BCC1 matrix phase and oxide phase are largely retained. This observation is consistent with the relatively high Ept of the Al-0 alloy, indicating that a relatively low anodic current density can be maintained over a broader potential range before a pronounced current increase and localized dissolution occur. The BCC2 phase appears to be the first region to undergo localized dissolution after the forward potentiodynamic scan, whereas the BCC1 matrix and oxide phase are largely retained. This result demonstrates phase-selective corrosion under the applied polarization condition, but does not by itself establish a phase-resolved corrosion potential difference. Therefore, within the same scan range, corrosion in the Al-0 alloy is relatively limited and mainly manifests as localized phase-selective dissolution.

In contrast, corrosion in the Al-0.2 alloy was concentrated mainly in the compound phase distributed along the matrix grain boundaries, whereas the BCC matrix showed almost no obvious dissolution. Compared with the Al-0 alloy, the area of dissolution increased markedly, and nearly all grain-boundary-associated compound regions around the matrix grains underwent corrosion to varying degrees. This behavior is consistent with the lower Ept of the Al-0.2 alloy, which indicates that pronounced anodic current increase and localized dissolution occurred at a lower potential during the forward scan. The result suggests that the boundary-associated compound regions are more susceptible to passive-film breakdown under the applied polarization condition.

For the Al-0.5 alloy, the Al-Zr-rich compound phase distributed along the grain boundaries was almost completely dissolved after corrosion, forming a continuous network-like corroded region together with numerous round corrosion pits. The distribution of the pits was highly consistent with the pre-corrosion locations of the Al-Zr-rich phase, indicating that these boundary-associated regions were preferentially susceptible to dissolution during the applied anodic scan. The lower Ept and narrower ΔEp of this alloy indicate that pronounced passive-film breakdown or localized dissolution occurred within a relatively narrow potential range. However, the present observations do not directly determine the local corrosion potential of the compound phase or quantitatively establish its potential difference from the matrix. In addition, some compound phases located within the matrix grains remained after corrosion, whereas those located at grain boundaries were almost completely dissolved. This result suggests that localized corrosion is governed not only by the phase composition but also by the spatial distribution of the phase. Grain-boundary regions generally possess higher defect density and stronger elemental segregation and are therefore more likely to act as electrochemically heterogeneous regions where passive-film breakdown and localized corrosion propagation preferentially occur. The increasing area fraction and interconnection of the boundary-associated Al-Zr-rich compounds with increasing Al-for-Ti substitution may further promote the formation of continuous localized-corrosion pathways.

The Al-1 alloy exhibited the most severe corrosion among the investigated compositions. After polarization, the Al-Zr-rich compound regions were almost completely dissolved, and numerous corrosion pits propagated into the surrounding matrix. The Al-1 alloy also exhibited the lowest Ept and the narrowest ΔEp, indicating the earliest occurrence of pronounced current increase and localized passive-film breakdown during the forward anodic scan. The more extensive and interconnected distribution of the Al-Zr-rich compound regions is therefore considered to provide a larger number of susceptible sites and continuous pathways for localized dissolution. This interpretation is supported by the combined polarization, quasi-in-situ SEM, and FIB observations, although phase-resolved local electrochemical measurements would be required to directly verify the potential difference between the compound regions and the BCC matrix. The preferential dissolution of the Al-Zr-rich regions indicates their greater susceptibility to localized attack under the applied polarization condition. This observation is consistent with microgalvanic corrosion associated with the multiphase microstructure, but does not directly establish the corrosion potential of the compounds relative to the BCC matrix. Local electrochemical measurements, such as scanning Kelvin probe force microscopy (SKPFM) or micro-electrochemical tests, would be highly desirable to directly measure the local Volta potential difference or corrosion potential.

The quantitative image analysis provides further support for the relationship between microstructural evolution and localized corrosion. With increasing Al-for-Ti substitution, the area fraction of the Al-Zr-rich compounds increases from 15.97% in Al-0.2 to 22.83% in Al-0.5 and 27.86% in Al-1, accompanied by a more interconnected distribution along matrix grain or interphase boundaries. These regions introduce a larger number of microstructurally heterogeneous sites and increase the spatial continuity of the preferential corrosion pathways. The increase in Al-Zr-rich compound area fraction is therefore consistent with the progressively more severe corrosion observed in the Al-containing alloys, including the preferential dissolution of boundary-associated compounds, the formation of interconnected corrosion regions, and the deeper inward propagation of corrosion in the Al-0.5 and Al-1 alloys. It should be noted that the area fraction of the Al-Zr-rich compounds does not by itself establish a difference in local corrosion potential between the compounds and the BCC matrix. Rather, it provides a quantitative measure of the amount of the phase that is susceptible to preferential dissolution. The combined evidence from phase distribution, quantitative area-fraction analysis, potentiodynamic polarization, quasi-in-situ SEM, and FIB cross-sectional observations is consistent with possible microgalvanic interactions, although it does not directly establish phase-specific electrochemical potentials.

The FIB cross-sectional results further reveal the depthwise propagation characteristics of corrosion (Figure 8). The corrosion depth in the Al-0 alloy is approximately 12 μm, indicating that although localized corrosion has occurred, its inward penetration is relatively limited. In contrast, the corrosion depths of all the Al-containing alloys are significantly larger and even exceed the FIB milling depth, indicating that with increasing Al content corrosion not only spreads over a wider surface area but also penetrates more deeply along the thickness direction. In particular, more numerous and larger voids and cavities are observed in the cross section of the Al-1 alloy, indicating more severe selective removal of the preferentially dissolved phase during corrosion. Considering that the Al-1 alloy contains a relatively large amount of Al-Zr-rich compound regions, their extensive distribution may provide more susceptible sites and promote localized dissolution along the surrounding matrix.

Taken together, the EIS, potentiodynamic polarization, quasi-in situ SEM, and FIB cross-sectional results indicate that the susceptibility to localized corrosion increases significantly with increasing Al content. Although a high Al content may improve the barrier property of the surface film under open-circuit conditions, the Al-induced Al-Zr-rich intermetallic compounds and electrochemical heterogeneity at grain boundaries markedly impair passive film stability during anodic polarization and intensify the tendency for galvanic corrosion. As a result, the corrosion potential shifts negatively, the corrosion current increases, and the passivation interval narrows. Therefore, the addition of Al does not improve the corrosion resistance of this alloy system under potentiodynamic polarization; instead, it promotes the formation of active phases and aggravates localized corrosion, thereby reducing the overall corrosion resistance.

It should be noted that there is an apparent difference between the EIS and potentiodynamic polarization results. EIS mainly characterizes charge transfer and ion-blocking properties of the initial passive film near OCP, and is therefore more sensitive to the integrity and compactness of the native oxide or early-stage passive film. By contrast, potentiodynamic scanning is more effective in reflecting film formation, dissolution, and breakdown during anodic polarization. Thus, the former represents the short-term barrier property of the film under near-equilibrium conditions, whereas the latter more directly characterizes the dynamic stability of the film under continuous polarization. For the alloys investigated here, a higher Al content may promote the formation of Al-rich oxides or multicomponent composite films under open-circuit conditions, thereby resulting in higher film impedance. At the same time, however, the Al-induced changes in phase constitution, elemental segregation, and local electrochemical heterogeneity can facilitate local dissolution and film destabilization under anodic polarization. Therefore, the EIS and potentiodynamic polarization results are not contradictory, but rather reveal the dual role of Al in the corrosion behavior of this alloy system from different perspectives.

### 3.3. Corrosion Mechanism

**Figure 9:**
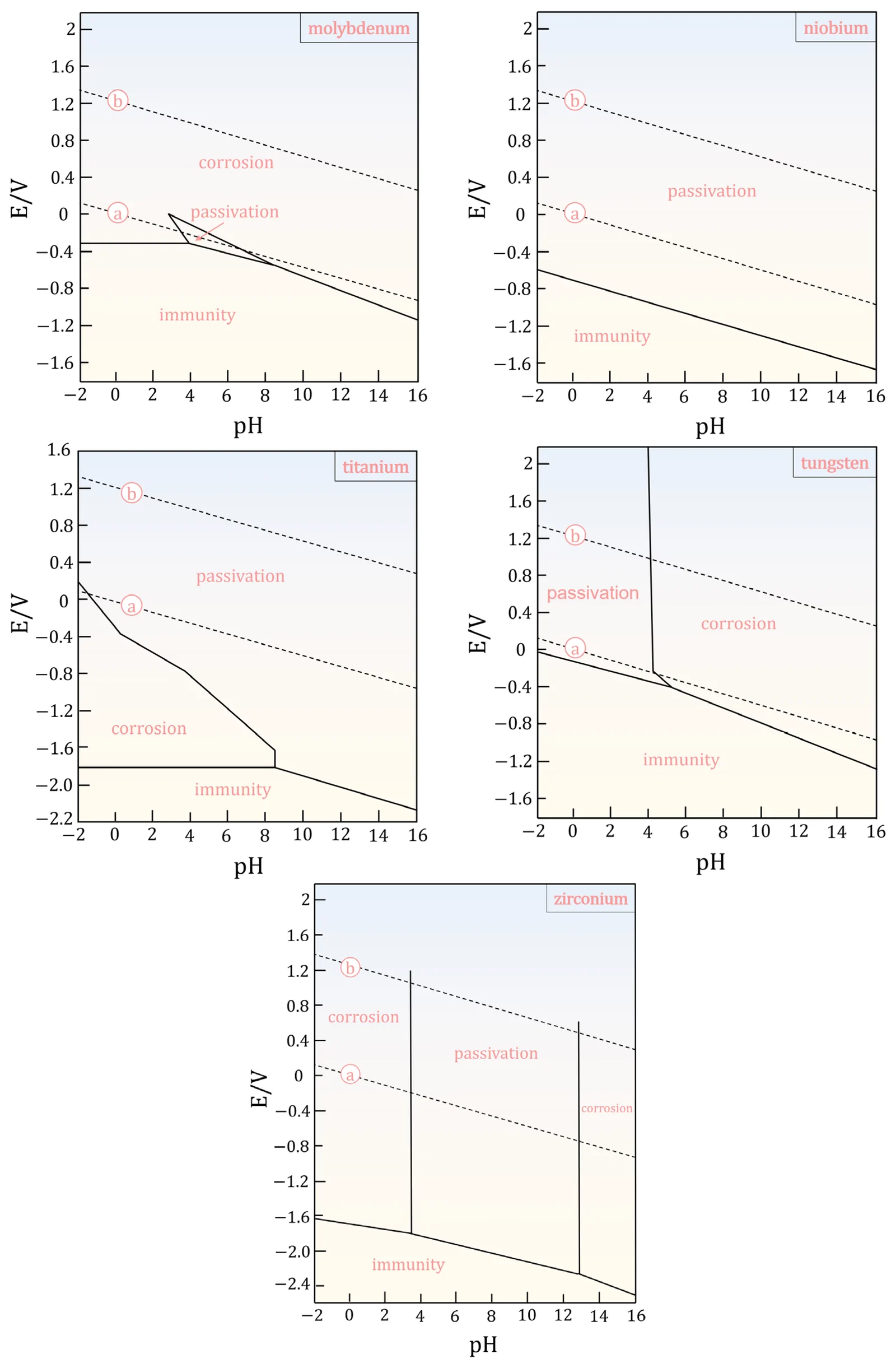
Pourbaix diagrams of Mo, Nb, Ti, W and Zr at standard condition (E/V vs. SHE). The dashed lines (a) and (b) represent the boundaries of water redox reactions, illustrating the stability region of water.

Pourbaix diagrams describe the thermodynamic stability of metals in a given environment as a function of potential and pH and are useful for analyzing the possible passivation behavior of alloying elements [37,38]. Based on the Pourbaix diagrams of the principal elements (Figure 9) and the electrochemical results obtained in near-neutral 3.5 wt.% NaCl solution, Nb-, Ti-, and Zr-containing oxides may contribute to passivation in this alloy system. The direct contributions of Mo and W to the formation of a stable passive film appear to be comparatively limited within the potential and pH range considered here. Among the potentially film-forming elements, Nb shows a relatively favorable thermodynamic stability within the relevant potential range and may therefore contribute to passive-film stabilization. However, this interpretation is based on thermodynamic analysis and electrochemical behavior rather than direct chemical characterization of the passive film.

The quasi-in situ corrosion results of the Al-0 alloy provide additional information concerning the relative corrosion susceptibility of the constituent phases. The BCC2 phase preferentially dissolves after the potentiodynamic scan, whereas the BCC1 matrix remains largely intact. This observation suggests that the BCC1 matrix has a relatively higher resistance to localized attack under the applied polarization condition. It does not, however, directly identify the passive-film composition or establish the specific contribution of individual oxides. Nb_2_O_5_ may be a possible stabilizing oxide species based on thermodynamic considerations; nevertheless, the passive-film composition was not directly measured in this study, and surface-sensitive analysis such as XPS is required to verify this interpretation.

With increasing Al-for-Ti substitution, the passivation potential of the Al-0, Al-0.2, and Al-0.5 alloys changes only slightly, whereas the passive-film breakdown-related potential decreases markedly. This indicates that the coupled compositional change has a relatively limited effect on the initial formation of the passive-like film, but reduces its stability at higher anodic potentials. The Al-for-Ti substitution, together with the composition-dependent sintering schedule, was associated with an increase in the formation and interconnection of Al-Zr-rich compound regions, thereby enhancing the electrochemical heterogeneity of the microstructure. Meanwhile, the accompanying decrease in Ti may reduce the contribution of Ti to the formation and stabilization of the oxide film on the BCC matrix. Therefore, the reduction in passive-film stability should be interpreted as the combined consequence of Al enrichment and Ti depletion rather than as the isolated effect of Al addition. The Al-Zr-rich regions were preferentially dissolved during the applied anodic scan, indicating greater susceptibility to local film breakdown or dissolution under these conditions. This behavior is consistent with the role of microstructural electrochemical heterogeneity in localized corrosion; however, the present measurements do not directly determine the local corrosion potentials of individual phases. Hence, the deterioration in corrosion resistance associated with Al-for-Ti substitution is manifested mainly by the increased amount and connectivity of preferentially dissolved Al-Zr-rich compound regions and enhanced boundary-associated corrosion, accompanied by a lower passive-film breakdown-related potential and more severe localized corrosion.

**Figure 10:**
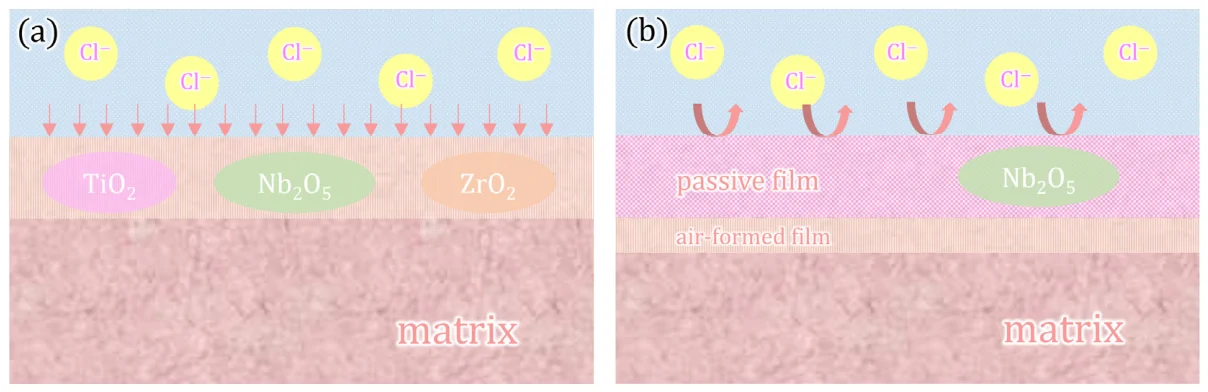
Schematic diagram of passivation film formation mechanism: (**a**) schematic of the initial surface state and the passive film formation process; (**b**) schematic illustrating the breakdown and localized dissolution mechanism of the passive film.

Based on the above analysis, a possible corrosion sequence during potentiodynamic scanning is proposed in Figure 10. A native oxide film may form upon exposure to air and may undergo reduction and restructuring during the initial polarization stage. In the passive region, Nb-containing oxides may contribute to film stability, potentially together with Ti- and Zr-containing oxides. These film-composition and evolution processes are proposed interpretations rather than directly observed results. At higher anodic potentials, preferential film breakdown or dissolution may occur in the Al-Zr-rich compound regions and their interfaces with the matrix, potentially assisted by microgalvanic interactions. Preferential dissolution of these regions can produce interconnected boundary-associated corrosion paths and inward corrosion propagation.

**Figure 11:**
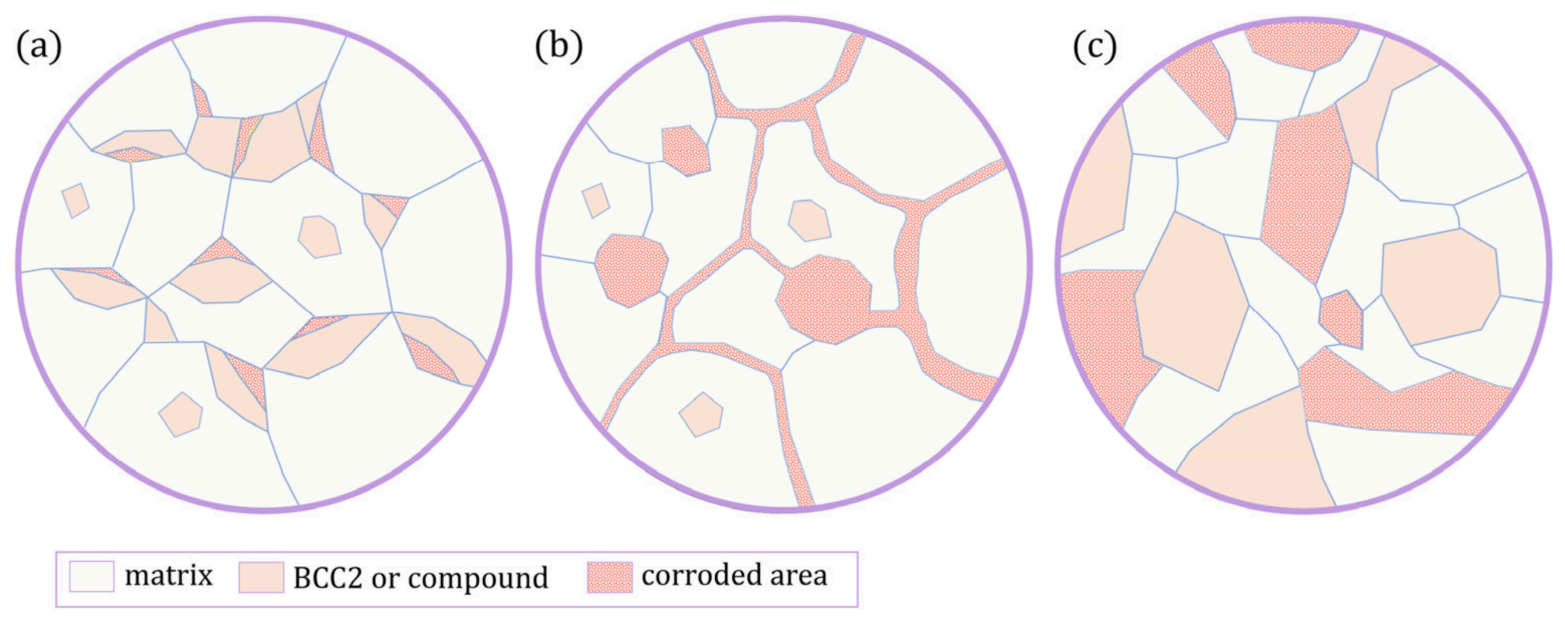
Schematic diagram of corrosion mechanism for alloys with different Al contents: (**a**) Al-0.2 alloy; (**b**) Al-0.5 alloy; (**c**) Al-1 alloy.

The corrosion pathways differ significantly with Al content (Figure 11). For the Al-0 and Al-0.2 alloys, the precipitate content is relatively low and the distribution is relatively discrete, so corrosion is mainly manifested as localized selective dissolution of the BCC2 phase or grain-boundary compound phase. For the Al-0.5 alloy, the Al-Zr-rich compounds form a continuous network along grain boundaries. Even though the matrix can still form a relatively stable passive film, this networked active phase destabilizes and dissolves first at lower potentials, thereby creating connected corrosion pathways. For the Al-1 alloy, the measured area fraction of Al-Zr-rich compounds increases further, providing more extensive regions susceptible to preferential dissolution and potentially promoting microgalvanic interactions. Corrosion therefore not only concentrates in the Al-rich phase regions, but also rapidly extends inward, ultimately leading to the most severe localized corrosion and cavity formation. Overall, the corrosion behavior of (Al_x_)Mo_0.8_NbTi_y_W_0.2_Zr alloys in near-neutral chloride-containing media may be interpreted in terms of passivation potentially involving Nb-containing oxides, together with local destabilization associated with the coupled Al increase and Ti decrease. The alloys exhibit passivation during polarization, whereas increasing Al-for-Ti substitution is associated with more extensive Al-Zr-rich compound regions and more severe localized dissolution under the applied anodic scan; microgalvanic interactions may contribute to this behavior.

Compared with previous corrosion studies on high-entropy alloys, the influence of Al on the passivation behavior of (Al_x_)Mo_0.8_NbTi_y_W_0.2_Zr alloys exhibits a pronounced composition dependence and a competitive mechanism. Previous studies have shown that in systems such as Al_x_CoCrFeNi and Al_x_CrFeMnNi, the addition of Al typically induces a phase transition from FCC to BCC/B2, aggravates elemental segregation, second-phase precipitation, and interphase boundary formation, and thereby increases local electrochemical heterogeneity, weakens passive film stability, and reduces corrosion resistance. In the present study, the decrease in low-frequency impedance modulus and film resistance at low Al contents is consistent with this trend, suggesting that under such conditions the enhancement of microstructural heterogeneity and microgalvanic effects dominates the corrosion process and is unfavorable for the formation of a stable and compact passive film. However, with increasing Al-for-Ti substitution, the impedance response of the alloy rises significantly, indicating that the role of Al is not limited to intensifying microstructural heterogeneity, but may also involve promoting the formation of a more stable Al-rich oxide film or multicomponent composite passive film at higher contents, thereby enhancing film barrier performance and overall corrosion resistance under open-circuit conditions. Therefore, unlike the commonly observed tendency in conventional high-entropy alloys where increasing Al continuously decreases corrosion resistance, the present results indicate that the role of Al in this RHEA is essentially determined by the competition between enhanced microstructural heterogeneity and improved film stability; the former dominates at low Al contents, whereas the latter begins to dominate the electrochemical response at higher Al contents.

### 3.4. Service Implication

The EIS and potentiodynamic polarization results reflect different aspects of corrosion behavior: the former characterizes the interfacial response near OCP, whereas the latter reveals increasing susceptibility to localized breakdown with increasing Al-for-Ti substitution. EIS mainly characterizes the impedance response of the surface film near OCP under small perturbation conditions, and is therefore more sensitive to the integrity and compactness of the native oxide film or early-stage passive film. Forward potentiodynamic polarization, by contrast, is used here to evaluate passive-film stability and susceptibility to localized breakdown under sustained anodic polarization; it does not directly evaluate repassivation ability. For the alloy system investigated here, a higher Al content may improve the barrier property of the surface film under open-circuit conditions, but during anodic polarization the Al-Zr-rich phases and grain-boundary regions are more prone to film destabilization and preferential dissolution under galvanic interaction. Consequently, potentiodynamic polarization is more sensitive to Al-induced localized corrosion.

Based on the present results, the corrosion behavior of the (Al_x_)Mo_0.8_NbTi_y_W_0.2_Zr alloys was evaluated under room-temperature conditions in 3.5 wt.% NaCl solution. Within this specific testing condition, the low-Al alloys exhibited relatively limited localized corrosion, which can be associated with their lower content of Al-Zr-rich compound phases and reduced microstructural electrochemical heterogeneity. In contrast, the Al-0.5 and Al-1 alloys showed more pronounced preferential dissolution of Al-Zr-rich phases, together with interconnected corrosion paths along grain or interphase boundaries and deeper corrosion penetration. These results indicate that, among the compositions investigated, the low-Al alloys exhibit better resistance to localized corrosion in room-temperature 3.5 wt.% NaCl solution, whereas increasing the Al-for-Ti substitution tends to aggravate localized corrosion under the same condition. This conclusion is limited to the experimental conditions used in this study and should not be directly extended to other chloride concentrations, temperatures, electrochemical loading conditions, or specific corrosion forms such as potential fluctuation-induced corrosion and crevice corrosion.

While this study elucidates the structure–corrosion relationship through potentiodynamic polarization and post-corrosion morphology, further electrochemical and microstructural characterizations would make this relationship more convincing. Specifically, cyclic polarization or potentiostatic tests would be highly beneficial to directly reveal the pitting and repassivation behavior, providing a quantitative evaluation of pitting susceptibility and film repairability. Moreover, quantitative characterization of the phase fractions, porosity, and grain-boundary density would enable a more rigorous correlation between microstructural parameters and localized corrosion kinetics, which should be the focus of future work.

## 4. Conclusions

The electrochemical corrosion behavior and corrosion mechanism of (Al_x_)Mo_0.8_NbTi_y_W_0.2_Zr refractory high-entropy alloys with different Al contents in 3.5 wt.% NaCl solution were investigated, and the following conclusions can be drawn:

(1) The sintered (Al_x_)Mo_0.8_NbTi_y_W_0.2_Zr alloys all possess a BCC matrix. With increasing Al content, the microstructure evolves from a combination of two BCC phases plus an oxide phase in the Al-0 alloy to a multiphase structure consisting of a BCC matrix and Al-Zr-based intermetallic compounds, and an ordered B2 phase further appears in the Al-1 alloy. Meanwhile, microstructural heterogeneity becomes more pronounced, with an increased amount and connectivity of Al-Zr-rich compound regions.

(2) In 3.5 wt.% NaCl solution, all alloys undergo passivation during potentiodynamic polarization. The passive film is inferred to contain Nb-, Ti-, and Zr-containing oxides based on the electrochemical results and thermodynamic considerations. Nb-containing oxides, possibly including Nb_2_O_5_, may contribute to passive-film stability. The addition of Al does not significantly alter the initial formation ability of the passive film, but markedly decreases its stability at higher anodic potentials, as evidenced by the continuous decrease in the passive-film breakdown-related potential and the pronounced narrowing of the passivation interval.

(3) With increasing Al-for-Ti substitution, the corrosion-sensitive regions shift from the localized BCC2 phase in the Al-0 alloy to grain-boundary compound phases in the Al-0.2 and Al-0.5 alloys, and further to large-scale preferential dissolution of Al-Zr-rich intermetallic compounds in the Al-1 alloy. The preferential dissolution of Al-Zr-rich regions is consistent with possible microgalvanic interactions with the BCC matrix, but does not establish phase-specific corrosion potentials or interphase potential differences. Corrosion propagates inward along grain and interphase boundaries.

(4) The corrosion behavior of (Al_x_)Mo_0.8_NbTi_y_W_0.2_Zr alloys in near-neutral chloride-containing media may be interpreted as passivation potentially involving Nb-containing oxides, together with localized destabilization associated with the coupled Al increase and Ti decrease. The increased amount and connectivity of Al-Zr-rich compound regions are associated with more severe localized corrosion under anodic polarization. Because x + y = 2, the observed corrosion degradation cannot be attributed to Al alone. The possible role of Nb-containing oxides remains to be verified by direct passive-film compositional analysis.

## Figures and Tables

**Table 1:** Chemical composition (at.%) of the different phases in as-sintered (Al_x_)Mo_0.8_NbTi_y_W_0.2_Zr alloys.

Alloy	Phase	Al	Mo	Nb	Ti	W	Zr	O
Al-0	BCC1	0	28.57	31.18	24.83	9.18	6.24	0
	BCC2	0	7.57	5.29	42.48	1.77	42.89	0
	Oxides	0	0.49	0.17	0.28	0.06	33.09	65.91
Al-0.2	Matrix (BCC)	3.50	16.12	24.01	30.76	9.69	13.75	2.17
	Compound	10.80	10.74	9.23	35.67	0.71	30.36	2.49
Al-0.5	Matrix (BCC)	4.33	19.72	21.37	31.01	9.79	12.25	1.53
	Compound	28.53	7.11	4.32	15.90	3.11	38.38	2.65
Al-1	Matrix (BCC)	10.92	18.96	24.51	24.42	6.71	12.12	2.36
	Compound1	21.97	10.65	7.35	16.21	2.66	37.30	3.86
	Compound2	33.82	6.45	10.48	9.12	0.63	36.96	2.54

**Table 2:** Nominal compositions of the investigated (Al_x_)Mo_0.8_NbTi_y_W_0.2_Zr alloys calculated from the initial powder stoichiometry (at.%).

Alloy	Nominal Formula	Al /at.%	Mo /at.%	Nb /at.%	Ti /at.%	W /at.%	Zr /at.%	Total /at.%
Al-0	Al_0_Mo_0.8_NbTi_2_W_0.2_Zr	0	16	20	40	4	20	100
Al-0.2	Al_0.2_Mo_0.8_NbTi_1.8_W_0.2_Zr	4	16	20	36	4	20	100
Al-0.5	Al_0.5_Mo_0.8_NbTi_1.5_W_0.2_Zr	10	16	20	30	4	20	100
Al-1	AlMo_0.8_NbTiW_0.2_Zr	20	16	20	20	4	20	100

**Table 3:** Fitted parameters of the selected equivalent circuit for the investigated alloys.

Sample	Al-0	Al-0.2	Al-0.5	Al-1
R_s_/Ω	20.31	18.83	18.92	19.04
R_1_/Ω·cm^2^	(1.42 ± 0.09) × 10^6^	(1.18 ± 0.06) × 10^6^	(1.17 ± 0.09) × 10^6^	(4.24 ± 0.12) × 10^6^
CPE_1_/μF·s^−n^·cm^−2^	3.00 × 10^−5^	3.14 × 10^−5^	3.58 × 10^−5^	2.04 × 10^−5^
n_1_	0.98	0.93	0.93	0.94
R_2_/Ω·cm^2^	(3.19 ± 0.07) × 10^4^	(1.76 ± 0.23) × 10^4^	(2.37 ± 0.17) × 10^4^	(3.20 ± 0.27) × 10^4^
CPE_2_/μF·s^−n^·cm^−2^	8.06 × 10^−5^	9.30 × 10^−5^	5.65 × 10^−5^	7.78 × 10^−5^
n_2_	0.85	0.93	0.92	0.91
χ^2^/10^4^	2.35	3.32	0.78	1.56

## Data Availability

The original contributions presented in this study are included in the article. Further inquiries can be directed to the corresponding authors.
